# Eugenol triggers CD11b^+^Gr1^+^ myeloid-derived suppressor cell apoptosis *via* endogenous apoptosis pathway

**DOI:** 10.1039/c7ra13499a

**Published:** 2018-01-19

**Authors:** Ying Ding, Zecheng Yang, Wensheng Zhang, Yuwei Xu, Yuanyuan Wang, Minghua Hu, Fangli Ma, Hanan Long, Ning Tao, Zhihai Qin

**Affiliations:** School of Basic Medical Sciences of Southwest Medical University Luzhou China; College of Life Science, University of the Chinese Academy of Sciences Beijing China; Department of Microbiology and Immunology, Shanxi Medical University Taiyuan China; Infinitus Chinese Herbal Immunity Research Centre, Infinitus China Company Ltd Guangzhou China; Key Laboratory of Protein and Peptide Pharmaceuticals, Institute of Biophysics, Chinese Academy of Sciences Datun Road No. 15 Chaoyang District Beijing China tao@ibp.ac.cn zhihai@ibp.ac.cn; Department of Pathology, The Affiliated Hospital of Southwest Medical University Luzhou Sichuan China longhanan@swmu.edu.cn; Department of Science and Technology, Southwest Medical University Luzhou Sichuan China

## Abstract

To study the effect and underlying molecular mechanism of eugenol on CD11b^+^Gr1^+^ myeloid-derived suppressor cells (MDSCs). The effect of eugenol on the inhibition of immortalized MDSC cell line MSC-2 and murine peritoneal macrophages was detected by MTT. Flow cytometry was used to detect the pro-apoptosis effect of eugenol on MDSCs. The expression levels of apoptosis-related proteins were detected by western blot. Eugenol has a selective inhibitory effect on MDSCs in a dose-dependent manner, which activates an endogenous apoptosis pathway, leading to apoptosis. Eugenol promotes the apoptosis of MDSCs *via* the intrinsic pathway.

## Abstract 2

To study the effect and underlying molecular mechanism of eugenol on CD11b^+^Gr1^+^ myeloid-derived suppressor cells (MDSCs).

## Graphical abstract

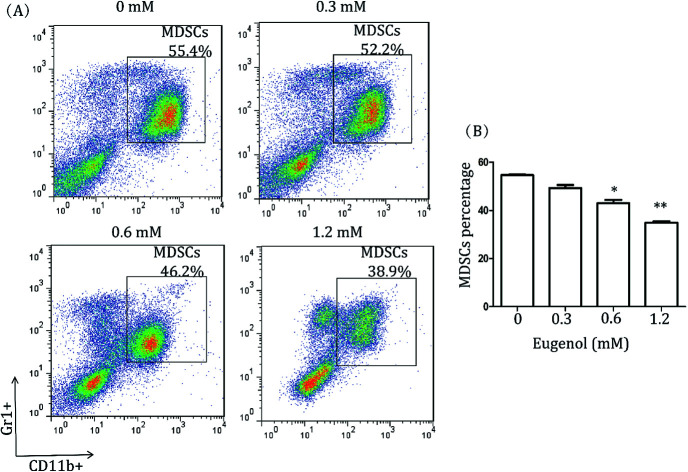

## 1. Introductions

Cancer is a major public health problem worldwide and is the main cause of death in most countries.^1–3^ However, conventional cancer therapies, including surgery, radiotherapy and chemotherapy, have faced impenetrable challenges due to their side effects and high recurrence rates.^4–6^ Therefore, safe and effective therapeutic strategies are urgently needed for treating malignant tumours.^7,8^

CD11b^+^Gr1^+^ myeloid-derived suppressor cells (MDSCs) are heterogeneous cells within the tumour microenvironment;^9,10^ they consist of myeloid precursor cells and immature myeloid cells, and they play a major role in the regulation of the immune response to tumours.^11,12^ Previous studies have demonstrated that MDSCs downregulate the innate and adaptive immune systems.^13,14^ As the primary immune suppressors of the tumour microenvironment, MDSCs have been shown to be effective targets for cancer therapy.^15,16^ Many molecules (for example, gemcitabine, docetaxel and 5-fluorouracil) targeting MDSCs have been demonstrated to have therapeutic effects in tumour treatment.^17–19^

Natural products are important for drug development, and many extractions of natural materials, Taxol and MPSSS, for instance, have strong anti-tumour features.^18–20^ Eugenol (4-allyl-1-hydroxy-2-methoxybenzene) is the main component of clove oil, which is often used as a preservative, analgesic and antimicrobial agent.^21^ Eugenol has extensive pharmacological effects, such as inhibiting the NF-κB pathways, participating in immune regulation and the anti-inflammatory response, blocking the activity of xanthine oxidase, affecting temperature-sensitive neuron discharge and antipyretic activities.^22–26^ Moreover, eugenol is used for animal anaesthesia, mosquito repellent and the promotion of drug permeation absorption. Eugenol shows potential for application in the prevention and treatment of certain tumours.^27–29^ However, whether eugenol can reduce immune suppression in the tumour microenvironment especially that caused by MDSCs, is unknown.

In this study, we explored the effect of eugenol on MDSCs. We found that eugenol effectively promoted the apoptosis of MDSCs and inhibited their immunosuppressive function. Our research reveals a novel use for eugenol in anti-tumour immunity, suggesting its potential use in anti-tumour therapy.

## 2. Materials and methods

### 2.1. Cell lines

Three immortalized cell lines were used in this study. MSC-2, generated from a retrovirus encoding the v-raf and v-myc oncogenes, had a macrophage-like phenotype and was provided by the Francois Ghiringhelli laboratory, Department of Medical Oncology, France. CT-26, a murine colon carcinoma cell line, was provided by Yang xin, Fu research group of the Institute of Biophysics, Chinese Academy of Sciences. MC-38 cells from a weakly immunogenic murine colon adenocarcinoma induced by the SC injection of dimethyl hydrazine in C57BL/6 mice were maintained in our research group. MSC-2, CT-26 and MC-38 cells were routinely cultured in DMEM high sugar medium (HyClone, USA) supplemented with 10% fetal bovine serum (PAN, Co. Germany), 1% penicillin and streptomycin. Cells were cultured at 37 °C in a fully humidified incubator equilibrated with 5% carbon dioxide (CO_2_).

### 2.2. The preparation of the primary cells

#### 2.2.1 Animals

BALB/c wild-type mice and TLR4 knockout C57BL/6 mice (18 to 22 g; 6 to 8 weeks old) were kept in rooms at 21 to 25 °C and 50% relative humidity with a 12 h light/dark cycle. Three mice were housed in each cage and provided with sterilized food and water. All animal care procedures were performed in accordance with the Guide for the US Department of Health and Human Services Publication Guide for the Care and Use of Laboratory Animals. Animal experiments were carried out in accordance with the Guidelines for the Care and Use of Laboratory Animals of the National Institute of Health, and were approved by the Biological Research Ethics Committee, Institute of Biophysics, Chinese Academy of Sciences.

#### 2.2.2 Preparation of murine peritoneal macrophages

The SPF male BALB/c mice (6 to 8 weeks old) received intraperitoneal injections of 1.5 mL starch broth. After three days, the peritoneal fluid was collected and cultured in DMEM complete medium. After 4 hours of incubation, the cells adhering to the bottom of the culture dish were determined to be peritoneal macrophages.

#### 2.2.3 Preparation of murine splenocytes

5 × 10^5^ CT-26 cancer cells and MC-38 cancer cells were injected subcutaneously in the flank of BALB/c wild-type mice and TLR4 knockout C57BL/6 mice, respectively. After 27 days, the splenocytes were isolated and treated with eugenol *in vitro*.

#### 2.2.4 Preparation of eugenol solutions

Eugenol was purchased from Beijing Beina Chuanglian Biotechnology Institute, China. It was dissolved in ethanol at different stock solutions and stored at 4 °C.

### 2.3. MTT assay

An MTT (3-[4,5-dimethylthiazol-2-yl]-2,5-diphenyl-tetra-zolium bromide) assay was used to evaluate the eugenol-induced cytotoxicity. Briefly, cells in the logarithmic growth phase were seeded into a 96-well culture plate at a density of 0.6 × 10^4^ cells per well in 100 μL, incubated with different doses of eugenol for 24 hours. Then 10 μL of the MTT solution (5 mg mL^−1^) was added and incubated for 4 hours before dissolving. Triple combine buffer (10% SDS, 5% isobutanol, 0.012 mol L^−1^ HCl, dissolved in distilled water) was added to dissolve the formazan crystal. The absorbance was measured at 570 nm using an absorbance microplate reader (BioTek, USA), and the half-maximal inhibitory concentration (IC_50_) values were determined.

Cell viability was calculated as follows: 100 × (absorbance of eugenol treated cells/absorbance of maximum MTT released control cells). Graph Pad Prism 5.0 was used to analyse the data.

### 2.4. Flow cytometry

#### 2.4.1 Apoptosis assay

MSC-2 cells were seeded into a 24-well plate at a density of 1 × 10^4^ per well, 24 hours later, eugenol at various concentrations were added. After incubation for an additional 24 hours, cells were collected and stained with Annexin V-FITC and PI (GenStar, China). Fluorescence was measured by FACS Caliber (Becton, Dickinson, USA) and the samples were analysed by Flowjo7.6 and GraphPad Prism 5.0.

#### 2.4.2 Detection of MDSCs in the splenocytes of tumour-bearing mice

Spleen cells isolated from tumour-bearing mice were seeded into a 96-well plate at a density of 5 × 10^5^ per well and treated with various concentrations of eugenol. After 24 hours of incubation, the cells were incubated with anti-Gr1 and anti-CD11b (BD Biosciences, USA) and detected by flow cytometry.

### 2.5. Western blot analysis of cell proteins

MSC-2 cells treated with a certain concentration. For various durations were harvested from a 6-well plate and then lysed in RIPA buffer (Beyotime, China) to isolate whole cell proteins. The cell extract containing proteins (30 μg) was separated on 12% SDS–polyacrylamide gels and electrophoretic ally transferred onto a nitrocellulose membrane (GE Healthcare, Milwaukee, USA). The membrane was blocked in 3% BSA in PBS-T (0.1% Tween-20) at 4 °C overnight and probed with the primary antibodies as follows: anti-p65 nuclear factor kappa B (NF-κB) monoclonal antibody, anti-β-actin monoclonal antibody, and anti-cytochrome C, anti-bax, anti-caspase 3, anti-BCL-2, anti-caspase 8, and anti-caspase 9 polyclonal antibodies (Cell Signalling Technology, US). After washing three times with PBS-T, HRP-conjugated goat anti-mouse or goat anti-rabbit IgG was used as the secondary antibody. Specific bands were visualized using the Chemiluminescence Imaging System (Clinx Science Instruments Co. Ltd, Shanghai, China).

### 2.6. Statistical analysis

All data are presented as the means ± SD (standard deviation) and evaluated by one-way ANOVA. *p < 0.05 was regarded as statistically significant. All of the experimental procedures were independently repeated at least three times.

## 3. Results

### 3.1. Eugenol reduces the proportion of MDSCs in splenocytes

**Fig. 1:**
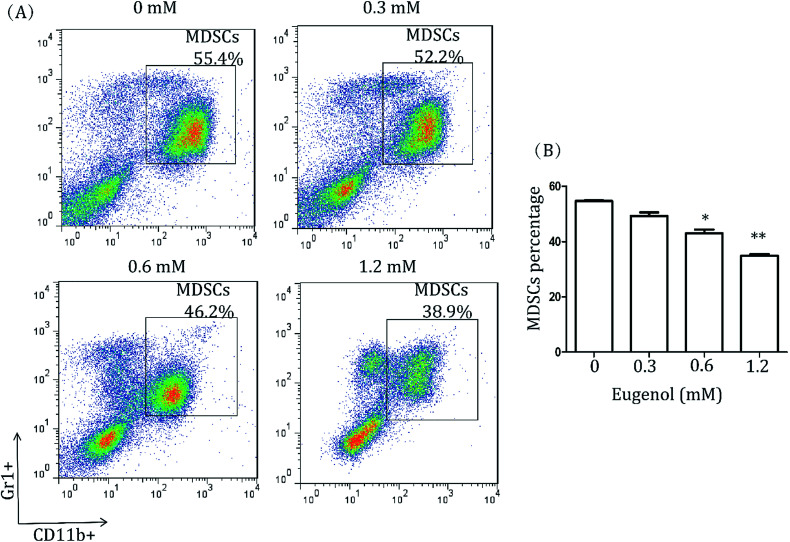
MDSCs in splenocytes of wild-type mice. (A) Splenocytes from CT-26 tumour-bearing mice (5 × 10^4^ per well in a 96-well culture plate) were treated with various concentrations of eugenol for 24 hours and then stained with fluorescent antibodies (anti-CD11b, anti-Gr1) and subjected to a fluorescence-activated cell sorting analysis on the flow cytometer (BD Calibur). Each group is set with four repeat wells. Data were analysed using FlowJo 7.6. (B) A bar chart was generated using Graph Pad Prism 5.0. *p < 0.05, **p < 0.01.

To study the effect of eugenol on MDSCs, spleen cells from CT-26 tumour-bearing mice were treated with different concentrations of eugenol and analysed by flow cytometry. As shown in Fig. 1, eugenol reduced the MDSCs (Gr1^+^, CD11b^+^) numbers in a dose-dependent manner and significantly suppressed MDSCs at a concentration of 0.60 mM.

### 3.2. Eugenol has an inhibitory effect on immortalized MDSC cell line MSC-2, and eugenol promotes the cell viability of macrophages near the IC_50_ concentration

**Fig. 2:**
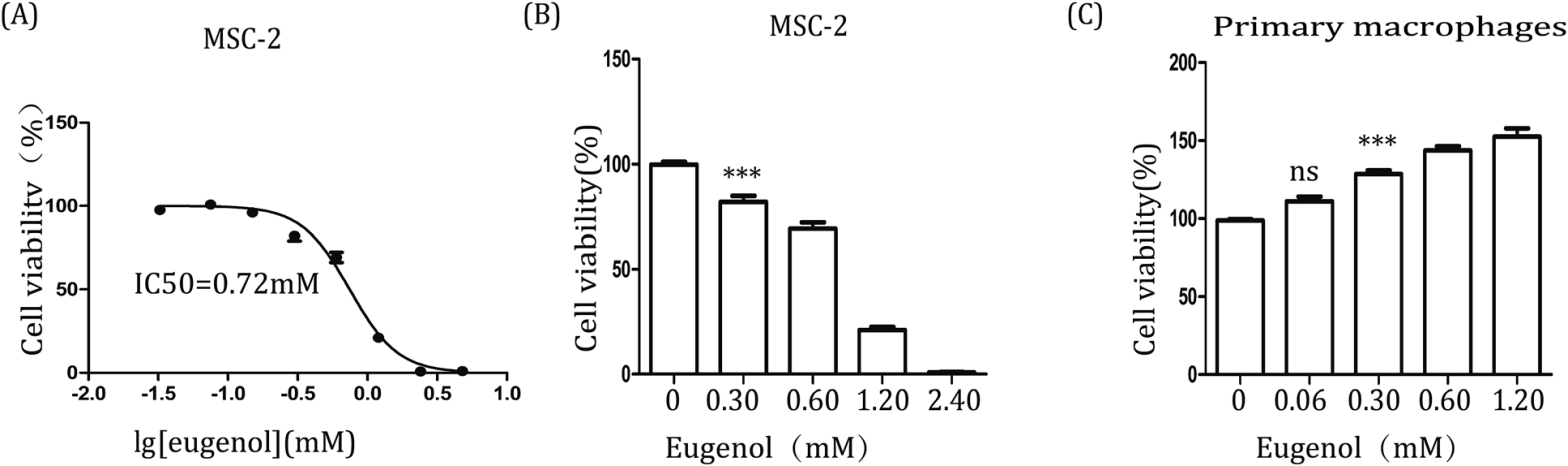
The effect of eugenol on MSC-2 cells and macrophages. (A & B) MSC-2 cells (0.6 × 10^4^ per well in 96-well culture plates). Each group is set with four repeat well; (C) macrophages (1 × 10^5^ per well in 96-well culture plates) were treated with various concentrations of eugenol for 24 hours. Cell viability was analysed by MTT assay. DMEM was used as the control. *p < 0.05, ***p < 0.001, and ns represents non-statistically significant differences.

Further, to examine the effect of eugenol on MDSCs, we first investigated the immortalized MDSC cell line MSC-2. After the cells were treated with eugenol at various concentrations for 24 hours, the concentration that resulted in ∼50% cell death (IC_50_) was determined to be 0.72 mM (Fig. 2A). Eugenol reduced the viability of MSC-2 cells in a dose-dependent manner and significantly affected cell viability at a concentration of 0.30 mM (*p < 0.05) (Fig. 2B). Interestingly, the MTT assay results also showed that the viability of macrophages (isolated from abdominal cavity of male BALB/c mice) was promoted when the cells were stimulated with eugenol (Fig. 2C).

### 3.3. Eugenol induces apoptosis in MSC-2 cells *via* the intrinsic pathway

**Fig. 3:**
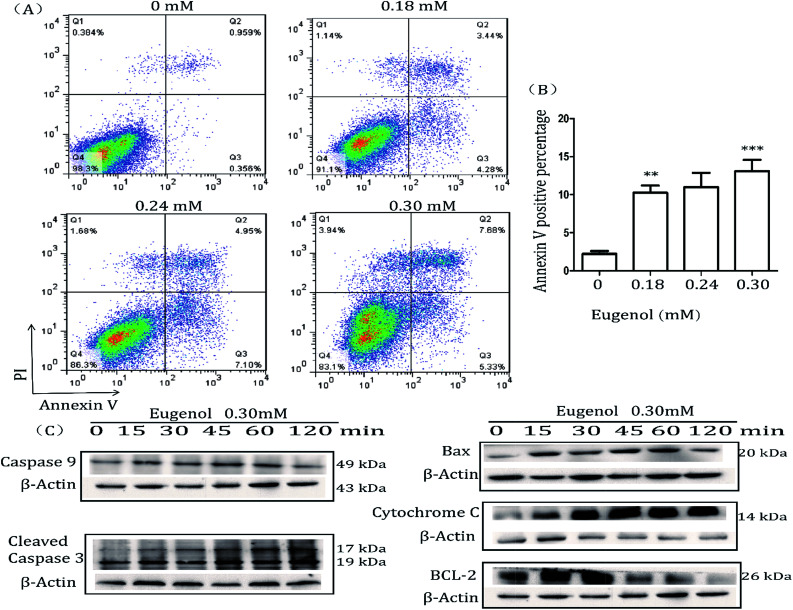
Flow cytometry analysis of cells stained with Annexin V-FITC and PI. (A) MSC-2 cells were treated with eugenol for 24 hours and then harvested and processed by Annexin V-FITC and PI staining followed by flow cytometry analysis. Each group is set with four repeat wells. The fluorescence pattern of Annexin V-FITC and PI-stained MSC-2 cells after 24 hours of treatment. (B) Percentages of Annexin V positive cells following different treatments. (C) Levels of bax, cytochrome C, caspase-9, cleaved caspase-3, BCL-2 and caspase-8 (no bands were detected) in total cell lysates were determined by western blotting. The expression of β-actin served as a control.

MTT results showed that eugenol decreased the proportion of living MSC-2 cells. We next investigated the mechanism underlying this phenomenon. The Annexin V-FITC/PI double-stained apoptosis detection assay revealed that the apoptosis rate of MSC-2 cells was significantly increased when cells were treated with increasing concentrations of eugenol (Fig. 3A and B). To investigate the molecular mechanism underlying eugenol-induced MSC-2 cell apoptosis, we performed a western blot to investigate the changes in expression of apoptosis-related proteins. As shown in Fig. 3C, the expression levels of caspase-9, cleaved caspase-3 and cytochrome C were upregulated in a time-dependent manner.

Caspase-8 was barely expressed, even when cells were treated for 120 min (data not shown). Given that caspase-9, cleaved caspase-3 and cytochrome C are all involved in the intrinsic apoptosis pathway, and caspase-8 is related to the extrinsic pathway, these findings suggest that eugenol induces the apoptosis of MSC-2 cells *via* the endogenous mitochondrial cytochrome C pathway rather than the extrinsic pathway. Bcl-2 was initially expressed, but expression decreased over time.

### 3.4. Eugenol could not significantly decrease the percentage of MDSCs in the tumour-bearing mice with TLR4 knockout

**Fig. 4:**
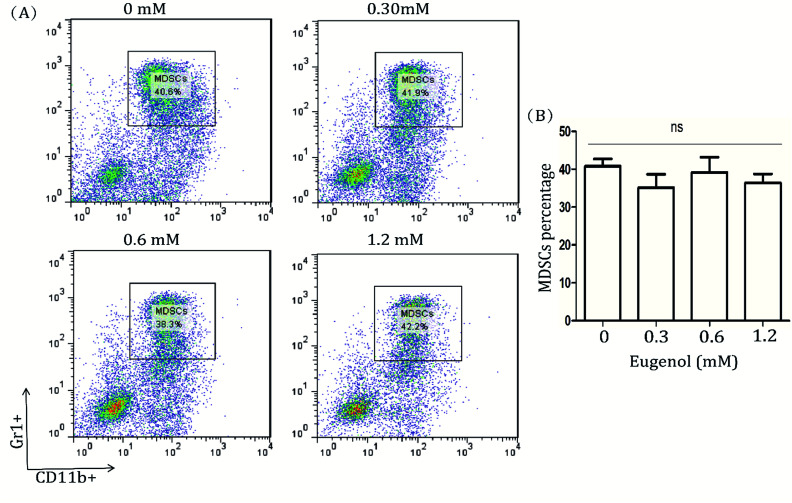
MDSCs in splenocytes of TLR4 knockdown mice. (A) Splenocytes from TLR4 knockout tumour-bearing mice (5 × 10^4^ per well in 96-well culture plates) were treated with eugenol at different concentrations for 24 hours and analysed by flow cytometry. Each group is set with four repeat wells. (B) The data analysis is shown in a bar chart. In addition, ns represents non-significant differences.

Detecting the spleen cells of other tumour-bearing mice, we found a different data. The results from eugenol-treated MDSCs isolated from TLR4 gene knockout mice indicated that eugenol did not significantly induce apoptosis in these MDSCs (Fig. 4A). That finding, taken together with the results of the wild-type BABL/c mice, indicated TLR was possible the receptor for eugenol on MDSCs (Fig. 1B and 4B).

## 4. Discussion

Many natural compounds have been used in cancer therapy to induce differentiation, inhibit function, and reduce the population of MDSCs.^16^ We investigated the percentage of MDSCs of splenocytes isolated from tumour-bearing mice. The results showed that the percentage of MDSCs in the spleen was decreased after treatment with eugenol (Fig. 1), suggesting that eugenol reduced the population of MDSCs. The MTT assay performed on the immortalized MDSC cell line MSC-2 confirmed this result, and the IC_50_ was determined to be 0.72 mM (Fig. 2A and B). Moreover, macrophages isolated from ascitic fluid demonstrated a visible proliferation when treated with eugenol, which may be evidence of an additional anti-tumour function of eugenol (Fig. 2C).

Apoptosis has been widely investigated for drug development and for elucidating anti-tumour mechanisms. Programmed cell death is launched from the intrinsic or extrinsic pathway, which are characterized by caspase-8 and caspase-9, respectively.^30,31^ In addition to the flow cytometry results showing that eugenol induced MSC-2 cell apoptosis (Fig. 3A and B), further experiments showed that the expression level of caspase-9 increased when cells were treated with eugenol (Fig. 3C). Taken together, these results suggested that eugenol activated the intrinsic apoptosis pathway in the immortalized MDSC cell line MSC-2. The loss of mitochondrial membrane potential results in the release of apoptosis-inducing proteins, such as cytochrome C, from the intermembrane space into the cytosol.^32,33^ The fate of cells undergoing apoptosis primarily depends on the ratio of antagonist molecules (Bcl-2, Bcl-xL, Mcl-1, and A1) to agonist molecules (Bax, Bak, Bcl-xs, and Bad).^34^ In this study, we found that the expression levels of cytochrome C and Bax increased, whereas the expression level of Bcl-2 decreased in a time depended manner in MSC-2 cells when treated with eugenol (Fig. 3C and D). This finding contributed to our conclusion.

Many studies have shown that the activation of the TLR4/NF-κB signalling pathway triggers apoptotic cascades.^35^ Our research demonstrated that eugenol did not decrease the ratio of MDSCs in the spleens of TLR4 knockout mice bearing colon tumours, suggesting that TLR4 is a possibly receptor involved in the promotion of apoptosis by eugenol. Further studies focusing on the detailed mechanisms and the role of TLR4 in this process are warranted.

**Fig. 5:**
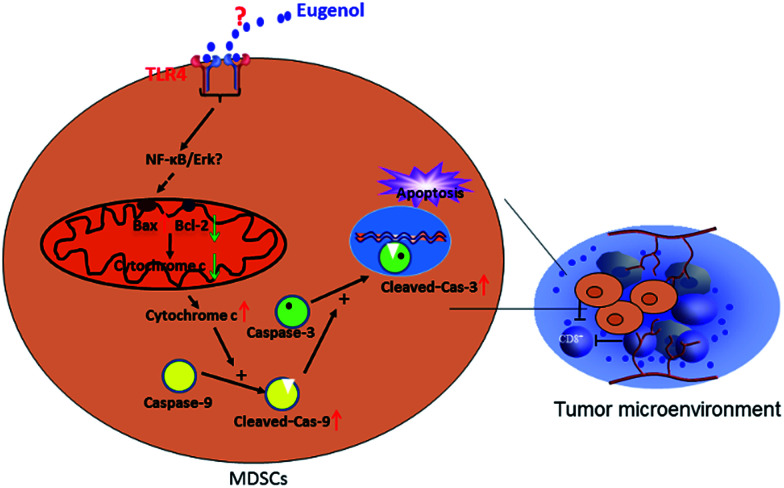
The apoptotic signalling pathway of MDSCs by eugenol.

In summary, our study demonstrated a strong anti-MDSC effect of eugenol in tumour immune therapy, suggesting the potential of eugenol as a prototype for designing and developing new anti-tumour medicine (Fig. 5).

## 5. Conclusions

In conclusion, we found that eugenol induces apoptosis in CD11b^+^Gr1^+^ myeloid-derived suppressor cells and promotes the viability of primary macrophages in mice, thereby mobilizing the body's ability to suppress tumour growth.

## Conflicts of interest

There are no conflicts to declare.

## Supplementary Material
